# Manufacturing of Large Size and Highly Transparent Nd:YAG Ceramics by Pressure Slip-Casting and Post-Sintering by HIP: An Experimental and Simulation Study

**DOI:** 10.3390/ma13092199

**Published:** 2020-05-11

**Authors:** Rémy Boulesteix, Cyril Chevarin, Rémy Belon, Alexandre Maître, Léo Cochain, Christian Sallé

**Affiliations:** 1 Institute of Research for Ceramics (IRCER), UMR CNRS 7315, University of Limoges, F-87068 Limoges, France; cyril.chevarin@umontpellier.fr (C.C.); belon@cilas.com (R.B.); alexandre.maitre@unilim.fr (A.M.); 2 Laboratory for Transparent Ceramics for Lasers (LCTL), University of Limoges, F-87068 Limoges, France; salle@cilas.com; 3 CILAS, F-45063 Orléans, France; 4 CERINNOV Group, F-87068 Limoges, France; lcochain@jlcoquet.com

**Keywords:** Nd:YAG, pressure slip-casting, simulation, laser

## Abstract

This study reports the fabrication of Nd:YAG (i.e., Neodymium-doped Yttrium Aluminum Garnet: Y_3-x_Nd_x_Al_5_O_12_) transparent ceramics of a large size by the pressure slip-casting forming technique. Colloidal suspensions of primary oxides (i.e., Y_2_O_3_, Al_2_O_3_, Nd_2_O_3_, and SiO_2_ used as sintering aid) were cast under pressure through a porous membrane. Cakes with a good microstructural homogeneity and mean pore diameter of 90 nm were obtained. Modeling of the pressure slip-casting process at the millimetric to centimetric scale based on a computational fluid dynamics simulation showed good agreement with experimental results in terms of the casting kinetics (i.e., cake thickness and fluid flow as a function of time) and cake permeability. As a result, it was possible to better manage pressure casting parameters in order to obtain large size and homogeneous green parts. Finally, transparent Nd:YAG ceramics sintered by vacuum sintering, followed by post-sintering treatment by Hot Isostatic Pressing (HIP), demonstrated laser slope efficiency (51.7%) and optical-to-optical efficiency (44%) with 130 mJ of output laser energy at 1064 nm equivalent to commercial single crystals.

## 1. Introduction

Ceramic processes usually use a liquid approach, i.e., involve the shaping of colloidal suspensions. Many different methods are used for shaping, such as slip-casting, pressure slip-casting, tape-casting, gel casting, centrifugal casting, etc. [1]. Among them, slip casting is an interesting casting technique, as it allows the manufacturing of large ceramic parts in a reduced amount of time. The casting of suspensions can be conducted in a natural way in a porous mold and/or with the assistance of pressure. In the first case, the porosity of the mold exerts a capillary pressure on the solvent that is extracted from the suspension at the surface of the mold. This leaves a layer of ceramic particles (called the “cake”) at the surface of the mold. To increase the casting rate, slip casting can be assisted by an additional pressure generally transmitted by a gas. This is known as “pressure slip-casting”, and is widely used in the industrial field to increase the production rates of ceramics [2].

In general, the flexibility of ceramic processes makes it possible to envisage the production of materials at a low cost, with a large size and/or doping gradient. They can thus respond to the challenges linked to the development of new laser amplifying media based on transparent ceramics, as described in the literature [3,4]. For example, the manufacturing of a segmented bar, with a gradual concentration of doping, makes it possible to minimize the longitudinal temperature differences and thus the stress gradients, according to the pumping direction in the laser cavity during the operation [5]. This type of architecture and others are easily achievable by the use of suitable ceramic shaping processes, followed by co-sintering of the elaborated composite parts [6,7,8,9,10].

However, transparent ceramics often have the disadvantage of exhibiting residual light scattering because of a too high level of residual porosity. Indeed, it has been shown that it is necessary to reach porosities lower than 10^−4^ vol.% for ceramics to achieve an optical and laser performance comparable to that of single crystals [11,12]. Many studies have focused on the use of ceramic processes to minimize the residual porosity of this type of material. In particular, it is generally accepted that the use of pure, homogeneous, and reactive nanopowders is required, as well as the use of sintering methods favoring densification of the material, such as natural vacuum sintering or pressure sintering methods like Hot Pressing (HP), Spark Plasma Sintering (SPS), and Hot Isostatic Pressing (HIP) [13,14,15,16].

On the other hand, the reactivity to sintering, that is to say, the capacity of a granular compact to densify, also strongly depends on its green microstructure, especially its pore size distribution [17]. This is largely conditioned by the powders and shaping technique used. The initial powders are chosen according to their particle size (often submicrometric), their morphology (spherical), and their purity [18]. Additionally, Rhodes [19] demonstrated that the presence of an inter-agglomerate porosity leads to differential sintering. Many studies have thus investigated the effect of shaping on the final material properties. Powder dry pressing is a simple shaping technique widely used in the industrial field. However, it is generally admitted that the microstructure of green compacts obtained by pressing is not homogeneous, contrary to liquid processes, which allow the particles to better rearrange during shaping [20]. For example, Mohammadi et al. [21] showed that the best transparency of YAG ceramics was achieved for samples shaped by slip-casting, which led to a narrow pore size distribution and high relative density of 64%. This study especially highlighted the critical effect of suspension formulation parameters, such as the dispersant content or solid fraction, on the optical quality of YAG ceramics. Esposito et al. [22] compared slip-casting to Cold Isostatic Pressing (CIP) and demonstrated that CIP also allows the production of powder compacts with a well-controlled and fine microstructure. In this case, samples obtained by CIP were more transparent than those produced by slip-casting owing to non-optimized powders and/or slurry formulation. Stevenson et al. [23] also compared dry shaping (uniaxial pressing followed by CIP) and liquid shaping (tape-casting followed by CIP). Their results showed that the pressed samples had a larger pore size, leading to a longer and higher sintering temperature being required to reach full transparency. All of these studies highlight that the choices relating to powders, shaping techniques, and associated parameters are essential to ensuring the homogeneity of the green part that controls, to a great extent, the material’s transparency after sintering treatment. The quality of the primary powder particle packing is thus more critical than the choice of a particular shaping process.

In this study, we focused on the use of pressure slip-casting for the manufacturing of transparent ceramics based on Nd:YAG. Slurry formulation based on primary oxide powder mixing in water has been studied. Modeling of the pressure-casting process was carried out by using the finite element method-based software, COMSOL Multiphysics^®^, and the simulated results were compared with those obtained experimentally. This study demonstrates that pressure casting is a shaping technique suitable for the manufacturing of transparent ceramics with a large size and laser quality equivalent to that of single crystals of the same composition.

## 2. Materials and Methods

### 2.1. Elaboration Process

**Figure 1:**
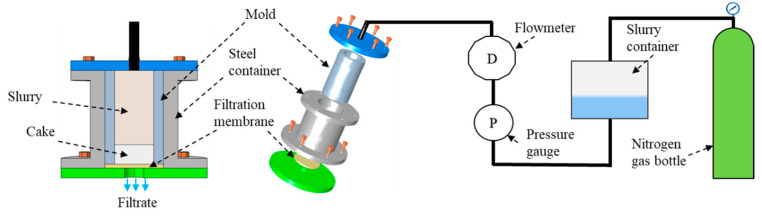
Scheme of the pressure slip-casting apparatus.

Commercial submicron α-Al_2_O_3_ (Ø < 0.5 µm; purity > 99.99%, Baïkowski, Poisy, France), Y_2_O_3_ (Ø < 0.5 µm; purity > 99.99%, Solvay, La Rochelle, France), 700 ppm wt. SiO_2_, and 1 at.% Nd_2_O_3_ (Ø < 1 µm; purity > 99.99%, Alfa Aesar, Kandel, Germany) were blended together in deionized water and ball-milled with corundum balls in order to obtain homogeneous aqueous slurries. Pellets were shaped by pressure slip-casting at 2 MPa under nitrogen gas pressure, as described in Figure 1. The CFD (Computational Fluid Dynamics) module of COMSOL^®^ software (version 5.3, COMSOL, Grenoble, France) was used to simulate the pressure slip-casting process.

After drying under air atmosphere, samples were calcined under air at T < 900 °C to remove organic residues. Reaction-sintering was conducted under vacuum in a tungsten mesh-heated furnace (*p* ≤ 10^−3^ Pa) at 1625 °C for 5 h, in order to obtain a 96%–99% theoretical density (i.e., 4.55 for YAG). Green bodies were placed in alumina crucibles with heating and cooling rates of 5 °C·min^−1^. In order to totally densify Nd:YAG samples, post-sintering treatment was applied by Hot Isostatic Pressing (post-HIP) at 1620 °C for 30 min under a pressure of 190 MPa of argon gas, as described elsewhere [13]. Samples were finally annealed under air at 1023 °C for 100 h.

### 2.2. Characterization

The viscosity of suspensions was measured by a rheometer (HAAKE MARS III, Thermo Fisher Scientific, Waltham, MA, USA) as a function of temperature or applied shear rate. Zeta potential measurements were performed for different suspensions loaded with 1 wt.% powder with an acoustosizer (IIS^TM^, Colloidal Dynamics, LLC, Ponte Vedra Beach, FL, USA). The pH was adjusted by adding 0.1 M solutions of HCl or NaOH to decrease or increase the pH, respectively.

After shaping by pressure casting, the pore size distribution in green compacts was determined by porosimetry by mercury intrusion (AutoporeIV 9510, Micromeritics, Norcross, GA, USA). The minimum pore diameter that could then be measured with such apparatus was around 3.5 nm. Green compacts were also characterized by high resolution X-Ray microtomography (Versa 500, Zeiss, Oberkochen, Germany) with a 150 nm spatial resolution.

After thermal treatments, the samples’ relative density was determined using the Archimedes method in ethanol. Sintered samples were then polished with 1 µm diamond paste and thermally etched under air at 100 K below the sintering temperature to investigate their microstructure by scanning electron microscopy (FEG-SEM Quanta 450, FEI, Thermo Fisher Scientific, Waltham, MA, USA).

For laser tests, all specimens were polished on both surfaces to reach an average roughness inferior to 0.2 nm, flatness near to λ/10, and parallelism < 10 arc seconds. The laser performance of specimens 13 mm in diameter and 2.5 mm thick was evaluated using a laser oscillation device pumped by a focalized beam of a 808 nm laser diode stack with a 5 Hz repetition rate. The pump pulse duration was 200 µs. The pump spot size in the sample was approximately 4 mm. No sample cooling was used and no antireflection coating was applied on the samples’ surface. Therefore, sample surfaces were orientated at Brewster’s angle from the optical axis of the laser cavity (cavity length of 0.29 m). The cavity was plane-parallel (two flat mirrors). The end-mirror (>99.8% reflection) and output coupler mirror (70% reflection) were flat and situated parallel to each other on both sides of the specimen. The laser beam energy was measured by a joulemeter detector (ED200, Gentec, Quebec City, QC, Canada). A 1.1 at.%-Nd:YAG single crystal (Czochralski grown, EOT GmbH, Idar-Oberstein, Germany) was used as the optical reference for all measurements.

### 2.3. Simulation of the Pressure Slip-Casting Process

The process for the pressure slip-casting of colloidal suspensions, and thus the flow, filtration, and deposition phenomena, can be observed at three different scales: microscopic, mesoscopic, and macroscopic [24]. A microscopic scale involves collections of particles whose movement and fluid flows between them are calculated. The Navier–Stokes equations are solved directly around the particles. This is called Direct Numerical Simulation (DNS). This approach is naturally suitable in the context of thin layer deposition generally encountered, for example, during waste and/or water filtration processes. At a mesoscopic scale, we consider the suspension (solvent + solid particles) a homogeneous medium. The mathematical approach used, called the Lagrangian approach, is based on an averaged formulation of the fluid, but for which the particle trajectories are calculated individually. In this way, the numerical method used for computing is called Discrete Element Method Computational Fluid Dynamics (DEM-CFD) or the Combined Continuum and Discrete Model (CCDM), and consists of describing the continuous phase (fluid) in a Eulerian way (average flow) and ensuring Lagrangian monitoring (individual) particles [25]. The calculations carried out at these first two scales, which are comparable to the size of the particles, make the determination of interesting characteristic quantities of the flow, such as the permeability, possible. Nevertheless, they are very demanding in terms of computing power and are time consuming, as it is necessary to describe a large number of pores and particles in motion [26,27,28]. Finally, the macroscopic scale corresponds to a larger scale that does not describe the motion of individual particles. The colloidal suspension and cake are assimilated as equivalent homogeneous media described by space-averaged equations and by a few parameters. Therefore, the flow phenomena in the fluid domain (slurry) and the porous domain (cake) are described by the Navier–Stokes and Darcy equations. To describe the deposition phenomena occurring during the pressure slip-casting process, the cake growth can be described by a simple deformation [29,30,31,32,33]. Additionally, the assumptions induced by this type of model considerably reduce the computation time and are well-adapted to the description of ceramic processes where large pieces with sizes often larger than 1 cm are expected to be manufactured. However, the physical characteristics of the slurry and cake are not easy to measure in-situ because of the high pressure, low flow rate, and generally weak repeatability of the tests.

As illustrated in Figure 1, the area of calculation inside the mold will be composed of two media: (1) a fluid medium consisting of the colloidal suspension, which flows freely, and (2) a porous medium or “cake” constituted by the nanoparticle deposit, inside which the filtered liquid (filtrate) flows. The filtrate (water) is considered to be a Newtonian fluid with a dynamic viscosity μ_filtrate_ = μ_eau_ = 1.01 × 10^−3^ Pa·s at 20 °C. The suspension is considered to be a non-Newtonian fluid, as described by Equation (6) in the next section.

The flow velocities of the suspension and filtrate were determined from mass flow rates measured experimentally using the device illustrated in Figure 1. It is worth noting here that the hydraulic resistance of the porous media (filtration membrane) ensuring filtration of the suspension was neglected for further calculations. The obtained values appeared sufficiently low (i.e., in the order of 10^−4^–10^−5^ m.s^−1^) to consider a creeping flow, also called “Stokes flow” (i.e., of laminar type). In this case, the filtrate flow through a deposit established by the stacking of particles on a porous surface, encountered in filtration, is conventionally modeled by Darcy’s law [34,35,36].

First, the flow of the suspension can be described using the Stokes equation (Equation (1)) [34], as follows:

(1) $$\frac{\partial }{\partial t}\left(\rho_{s}\vec{v}\left(t\right)\right)=-\nabla p+\nabla .(\overset{=}{\tau )}+\rho_{s}\vec{g}\;,$$

where *t* is the time (in s), *ρ_s_* is the volume mass of the suspension (in kg·m^−3^), $\vec{v}\left(t\right)$ is the velocity vector, ∇ is the Nabla operator ($\nabla G=\left(\frac{\partial G}{dx},\frac{\partial G}{dy},\frac{\partial G}{dz}\right)$ in Cartesian coordinates), *p* is the static pressure (in Pa), $\overset{=}{\tau }=\mu_{s}\left[\left(\nabla \;\vec{v}+\nabla \;{\vec{v}}^{T}\right)\right]$ is the viscous stress tensor, $\mu_{s}$ is the viscosity of the suspension (in Pa.s), and $\vec{g}$ is the gravity vector (in m·s^−^²).

Second, the flow of the filtrate in the cake can be described using a Darcy–Brinkman flow regime generalized [37] by Equation (2):

(2) $$\frac{\partial }{\partial t}\left(\rho_{f}\vec{v}\left(t\right)\right)=-\nabla p+\nabla .(\overset{=}{\tau )}+\rho_{f}\vec{g}-\frac{\mu_{f}}{k}\vec{v}\left(t\right),$$

where *μ_f_* is the dynamic viscosity of the filtrate (in Pa·s), and *k* is the permeability of the cake (in m²). In the case of such a low flow rate, the permeability *k* can be defined as a function of the cake microstructure by Ergun’s law (Equation (3)) [38,39,40]:

(3) $$k=\frac{\varepsilon^{3}d_{p}{}^{2}}{150{\left(1-\varepsilon \right)}^{2}}$$

where *d_p_* is the mean diameter of pores in the cake (in m) and *ε* is the total porosity fraction (0 < *ε* < 1).

From the law of conservation of matter at the cake/suspension interface, we can define by Equation (4) the speed of the mobile interface corresponding to the surface of the deposit:

(4) $$\vec{v_{s}}\left(t\right)=-\frac{f_{v}}{\left(1-\varepsilon \right)\;}\;\vec{v}\left(t\right),$$

where *f_v_* is the solid volume fraction of the suspension (0 < *f_v_* < 1) and $\vec{v}\left(t\right)$ is the suspension velocity vector at the cake/suspension interface at a given time (m·s^−1^). From Equation (4), it is thus possible to determine the growth kinetics of the cake. It is worth noting here that the model can be self-consistent and predictive if all parameters are known. In our case, the only parameter that we have let vary in the software is the permeability.

## 3. Results and Discussion

### 3.1. Green Body Formed by Pressure Slip-Casting and Characterization

**Figure 2:**
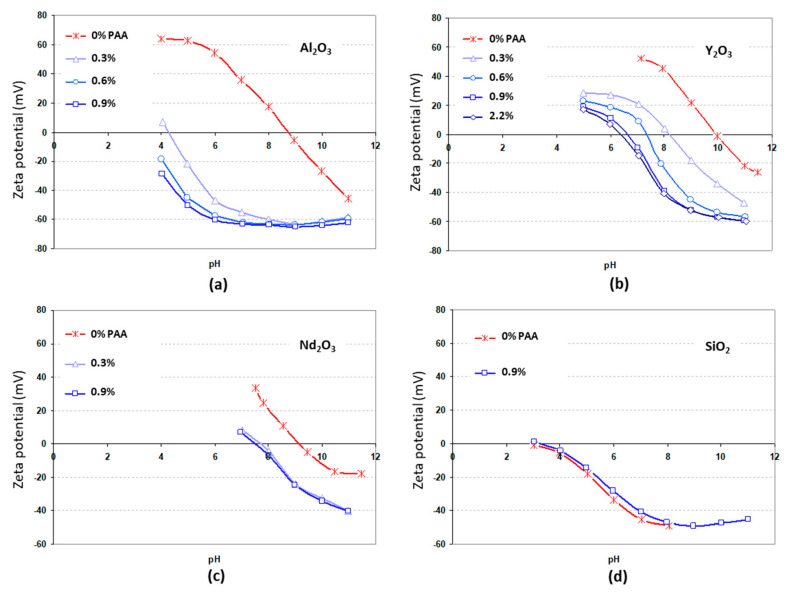
Evolution of the zeta potential of primary oxide (Al_2_O_3_, Y_2_O_3_, Nd_2_O_3_, and SiO_2_) aqueous suspensions as a function of the pH.(**a**) Al_2_O_3_ (**b**) Y_2_O_3_; (**c**) Nd_2_O_3_; (**d**) SiO_2_.

A high concentration and low viscosity are required for the casting of ceramic slurry in the pressure slip-casting process. For concentrated aqueous suspensions, a low viscosity is only achieved for well-dispersed particles with a high electrical charge (i.e., for Zeta potential, absolute values higher than 30–40 mV) at their surface. For this study, the influence of ammonium polyacrylate (PAA) addition on powders’ zeta potential in aqueous suspensions has thus been considered. In that case, suitable dispersion is ensured by electrosteric repulsion forces, owing to ionized PAA grafted to the surface of metal oxide particles (i.e., Al_2_O_3_, Y_2_O_3_, Nd_2_O_3_, and SiO_2_). In order to confirm this result, the zeta potential of suspensions was measured by acoustophorometry, as shown in Figure 2. This figure shows the evolution of zeta potential as a function of the pH of aqueous suspensions with different formulations. According to these results, it is shown that the three oxides Al_2_O_3_, Y_2_O_3_, and Nd_2_O_3_ have a large positive Zeta potential, even without the addition of PAA at pH < 7. Nevertheless, SiO_2_ presents a strongly negative charge for this pH. This can be explained by the isoelectric point (i.e., pH with zeta = 0 noted IEP) of the respective oxide surface. The Al_2_O_3_, Y_2_O_3_, and Nd_2_O_3_ surfaces display basic behavior (i.e., IEP = 9–10), while the SiO_2_ surface exhibits acidic behavior (i.e., IEP = 3). In our study, a very low content of SiO_2_ nanopowder was used (i.e., 700 wt. ppm) as sintering aid. Therefore, using pH = 7 would lead to heterocoagulation between SiO_2_ and other oxides and probably to lower homogeneity of SiO_2_ compared to PAA addition combined with pH > 9. Moreover, the viscosity of suspensions obtained without PAA addition at pH = 7 was still high (not measurable with the device used in this study and thus not suitable for casting), probably because the steric part of electrosteric repulsion forces induced by PAA is very significant. As a result, at least 1 wt.% of PAA should be added to the suspensions to ensure a highly negative zeta potential and good repulsion. Moreover, the pH value should be equal to or higher than 9. In such a case, a strongly negative zeta potential (i.e., <30 mV) is observed for all primary oxides studied here. As a result, the value of pH was fixed to 9 for all viscosity measurements, i.e., nearly the optimum value to ensure a highly negative zeta potential.

According to these results, aqueous suspensions of stoichiometric Y_2_O_3_/Al_2_O_3_ mixtures (56.6 wt.% Y_2_O_3_ and 43.4 wt.% Al_2_O_3_) with various solid loadings were elaborated at a pH value fixed at 9 to ensure good electrosteric repulsion between particles. First, the rheological behavior of suspensions with 1.3 wt.% of PAA was studied as a function of the solid loading (i.e., the volume fraction of particles in suspensions) and shear rate. Figure 2 shows the evolution of suspensions’ viscosity as a function of the shear rate $\dot{\varepsilon }$ with values between 100 and 700 s^−1^. Suspensions present different rheological behavior, depending on the solid loading. For solid loading *f_v_* < 25 vol.%, the results are in accordance with a shear thickening behavior, i.e., the viscosity increases with the shear rate. Such behavior is often encountered for concentrated suspensions or suspensions containing large agglomerates where particle or agglomerate interactions increase the apparent viscosity when the shear rate increases ([41] page 277–310). For *f_v_* = 25 vol.%, the suspension presents Newtonian rheological behavior with a roughly constant viscosity. The viscosity *η* of Newtonian suspensions can be expressed as follows (Equation (5)):

(5) $$\eta =\dot{\frac{\varepsilon }{\tau }}$$

where *τ* is the shear stress and $\dot{\varepsilon }$ is the shear rate. For *f_v_* > 25 vol.%, the rheological behavior of suspensions is pseudo plastic, with a shear thinning character that can be described by Shandraw’s law (Equation (6)):

(6) $$\mu_{susp}=\mu_{\infty }+\sigma_{0}\left(1-e^{-b\dot{\gamma }}\right),$$

where *σ_0_* is the yield stress, *μ_∞_* is the asymptotic dynamic viscosity at an infinite shear rate, *b* is a constant, and $\dot{\gamma }$ is the shear rate. For a Y_2_O_3_ + Al_2_O_3_ colloidal suspension with 1.3 wt.% of PAA and 30 vol.% concentrated, the parameters were determined as follows: *σ_0_* = 1.1 Pa, *μ_∞_* = 13 mPa·s, and *b* = 0.037 s. Shear thinning behavior is generally encountered in suspensions containing non-attracting anisotropic particles where particles interact with each other. The presence of a yield stress *τ_y_* to initiate flow can be attributed to a linkage of bonded particles in suspensions, i.e., with no shear rate suspensions, which should present a gel structure. This can be a disadvantage for the use of the slip-casting process (with or without pressure) because the flow rates are generally very low. Therefore, the casting rate can be very limited. This point will be discussed in the next section of the study. The change of suspensions’ rheological behavior as a function of solid loading can be summarized as follows: For low solid loading, large agglomerates (>1 µm) of nanoparticles interact with each other more and more when the shear rate increases, leading to an increase of the apparent viscosity (that remains very low and <10 mPa·s in any case). For high solid loading, suspensions present a gel structure due to a weak linkage of nanoparticles and/or agglomerates in such concentrated media. This leads to the presence of a yield stress *τ_y_* to initiate flow and to a lower apparent viscosity when the shear rate increases.

**Figure 3:**
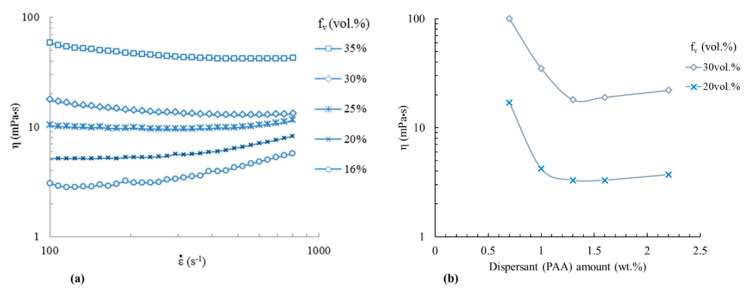
Evolution of suspensions’ viscosity at pH = 9 as a function of sharing stress for various particle volume fractions (f_v_) for 1.3 wt.% of ammonium polyacrylate (PAA) (**a**) and evolution of the viscosity at $\dot{\varepsilon }$ = 100 s^−1^ as a function of the dispersant amount for f_v_ = 20 and 30 vol.% (**b**).

The effect of increasing the dispersant amount at a given slurry concentration on the viscosity is given in Figure 3b. According to these results, the classical evolution of viscosity is observed to be at a minimum when the dispersant amount increases. The minimum obtained for both volume fractions (20 vol.% and 30 vol.%) is around 1.3 wt.% of PAA.

**Figure 4:**
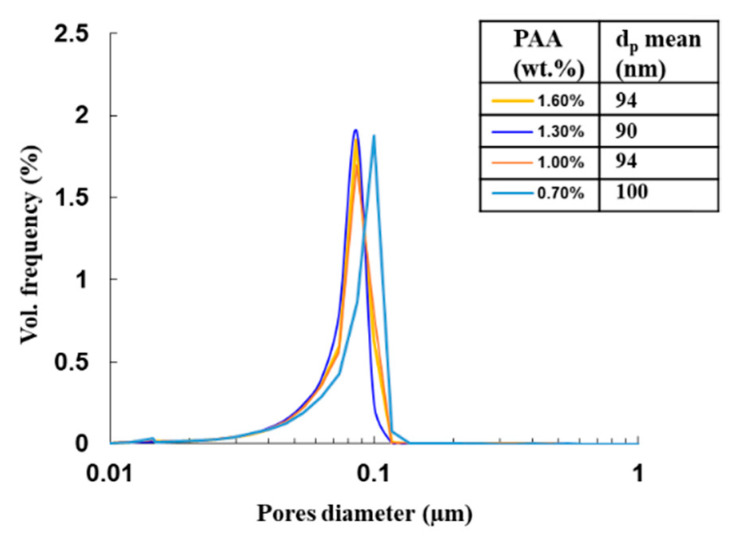
Evolution of the green compact pore size distribution as a function of dispersant amount. Corresponding *d_p_* mean values implemented in the model are given in the embedded table.

After shaping by pressure slip-casting at 30 MPa and drying, green compacts of 12 mm in thickness and 80 mm in diameter were characterized by porosimetry by mercury intrusion, in order to obtain the pore size distribution, as exposed in Figure 4. Measurements were conducted on suspensions with the pH fixed to 9 and various amounts of dispersant from 0.7 to 1.6 wt.%. In all cases, the pore size distribution is very narrow and centered around 80–100 nm, depending on the amount of PAA. Small variation in the pore diameter is evidenced and the results show a minimum pore size distribution for the dispersant amount of 1.3 wt.%, i.e., the amount leading to the minimum slurry viscosity (Figure 3b).

**Figure 5:**
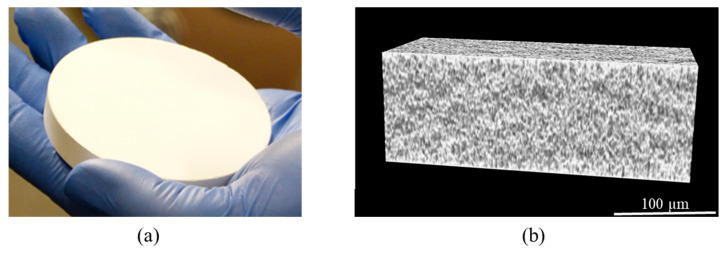
View of a green compact of Nd:YAG in the form of a pellet with a 80 mm diameter and 12 mm thickness obtained by the pressure slip-casting of suspensions with 30 vol.%, 1 wt.% of PAA, and pH = 9 (**a**) and a corresponding micrograph obtained by X-ray microtomography (**b**).

Furthermore, the green compacts obtained from suspensions with 30 vol.%, pH = 9, and 1.3 wt.% of PAA were characterized by X-ray microtomography and the result is shown in Figure 5. The microstructure appears to be homogeneous, with a very small pore size (i.e., <500 nm in diameter), in accordance with previous porosimetry analyses (Figure 4). As a conclusion, pressure slip-casting allows green compacts with a large size and a very homogeneous and fine microstructure to be obtained.

During simulations, the agreement between the permeability values as the model fitting parameter and permeability obtained from Equation (3) and the experimental measurement of porosity characteristics (ε and *d_p_*) of the cake was checked. The results obtained for the two sets of pressure slip-casting experiments conducted at a pressure of 3 × 10^6^ Pa and 1 × 10^6^ Pa are summarized in Table 1. All suspensions were elaborated with optimized parameters of 30 vol.% and pH = 9. Only the PAA amount was varied to control the viscosity parameter. First, these data show that a relative decrease of only 4% of the cake porosity and 5% of the mean pore diameter is observed when the pressure increases from 10 MPa to 30 MPa. As a result, this increase of pressure slightly decreases the permeability of the cake. The cake compressibility should be low, i.e., the microstructural features of the cake (pore size and porosity) should not be strongly dependent on the applied pressure in these conditions. Second, the set of data in Table 1 shows that suspensions’ viscosity (via the PAA content) has a strong influence on the microstructural features of the cake. This leads to significant variations in the permeability value calculated by Ergun’s law (Equation (3)), which increases from 2.7 × 10^−17^ m^2^ to 8.1 × 10^−17^ m^2^ when the viscosity increases from 18 mPa·s to 100 mPa·s. This can be explained by the easier rearrangement of powder particles when the viscosity decreases, i.e., when PAA reaches its optimum concentration, as exposed in Figure 3b.

**Figure 6:**
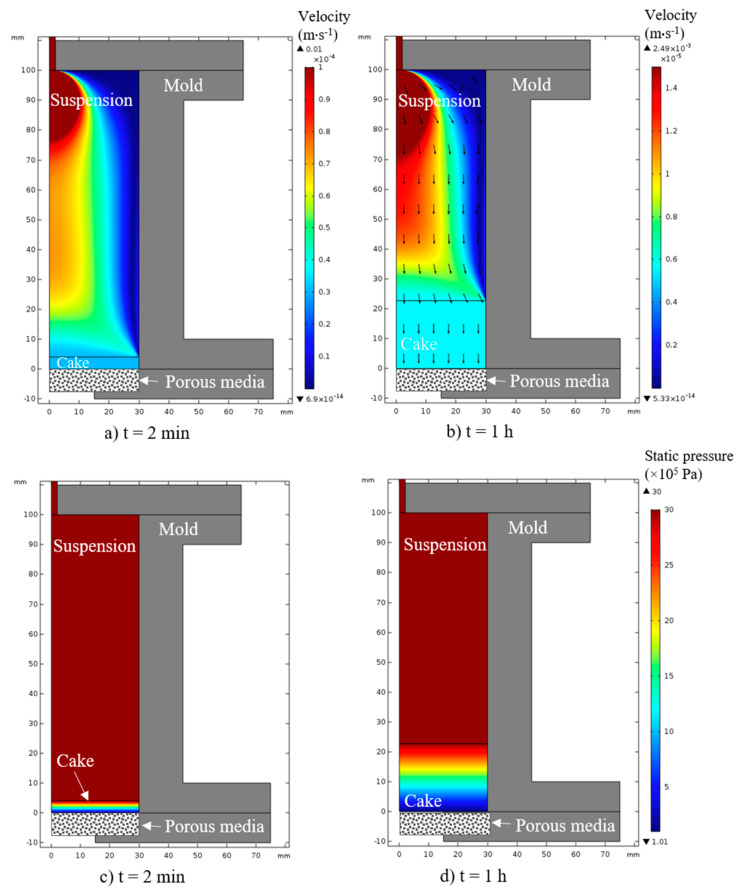
View of the pressure slip-casting system with the simulation result showing the liquid velocity field (magnitude and direction) (**a**,**b**) or static pressure (**c**,**d**) at different times of the process: (**a**,**c**) 2 min and (**b**,**d**) 1 h. Final cake thickness is 22 mm after 1 h filtration at a pressure of 3 × 10^6^ Pa.

Finally, the data in Table 1 shows that a good agreement is obtained between the two sets of permeability values. This is a first validation of the model developed in this study. From these calculations, the model allows the velocity field of the suspension and the filtrate, as well as the static pressure in the entire simulated zone (i.e., suspension and cake in the mold), to be described and predicted. The results obtained for a suspension with 1 wt.% of PAA, pH = 9, and 30 vol.% concentrated are illustrated in Figure 6 in a 2-D axisymmetric computational domain.

The flow velocities are very low and decrease during the formation of the green part as the latter creates hydraulic resistance to the flow. These velocities are in the order of 10^−4^ m∙s^−1^ at *t* = 2 min and 10^−5^ m.s^−1^ at *t* = 1 h, which is in agreement with a Stock-type laminar flow. In addition, a velocity gradient exists from the wall to the center of the device. This is because the flow velocities were fixed to zero at the surface of the walls of the mold.

The numerical model is also capable of predicting pressure fields at any point in the mold and over time. The pressure field is homogeneous at any time in the suspension, as illustrated in Figure 6c,d. Within the cake, a pressure gradient from 3 × 10^6^ to 1 × 10^5^ Pa is established from the surface of the cake (suspension side) to its center. It was supposed in the model that the outlet is subjected to atmospheric pressure. During the growth of the cake, the pressure gradient extends over all of its length and therefore becomes weaker.

**Figure 7:**
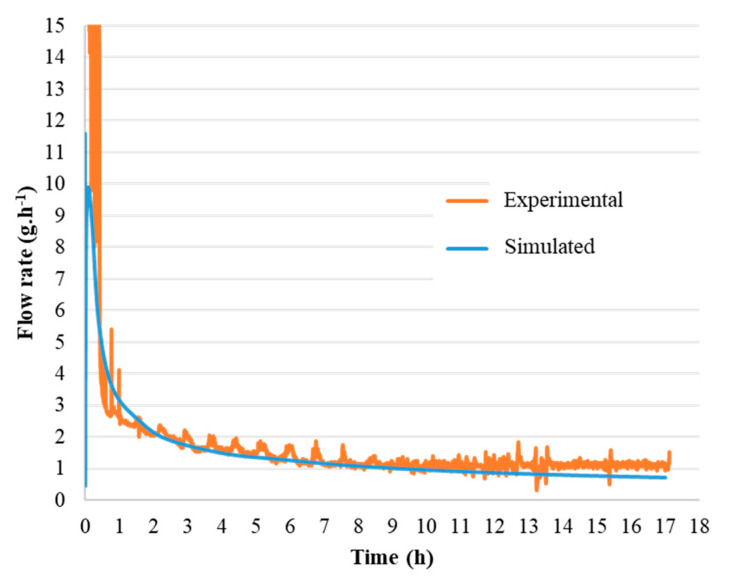
Comparison of the experimental and simulated injected mass flow rate of the suspension under a pressure of 3 × 10^6^ Pa for a cake with a 10 mm diameter.

The simulator is also able to calculate the injected mass flow rate of the suspension. Figure 7 compares the experimental inlet mass flow rate measured using the flow meter and simulated during the shaping of a cake with a 10 mm diameter. The simulated flow curve looks very similar to the experimental one and is almost superimposed on the latter. The differences observed remain in the measurement uncertainty, which once again makes it possible to validate the model under these experimental conditions.

**Figure 8:**
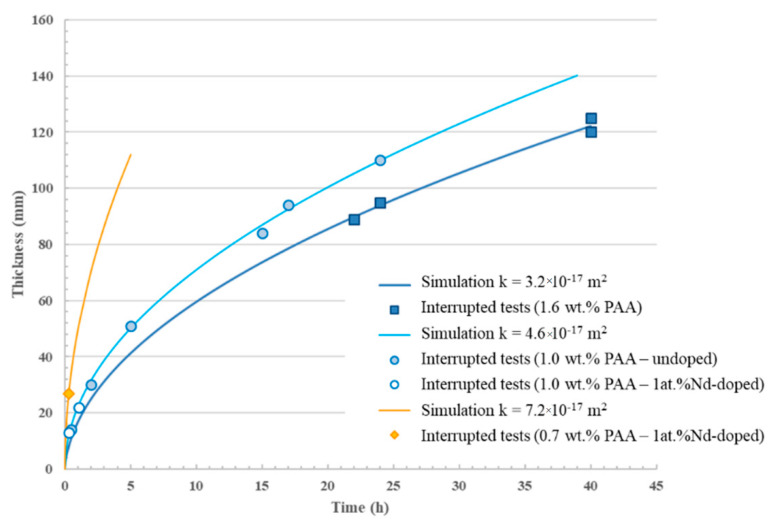
Evolution of cake thickness as a function of time at a pressure filtration of 3 × 10^6^ Pa for two different permeabilities showing good agreement between experimental points (stopped tests) and the simulation.

To validate the simulation results, YAG-based green compacts were shaped by pressure slip-casting under a pressure of 3 × 10^6^ Pa. The formulation of the suspensions used is presented in Table 1. The PAA amount has been varied to produce varying viscosity and corresponding filtration kinetics. Varying the viscosity resulted in slightly different cake porosity characteristics and thus permeability, as exposed in Table 1. The thickness of the parts obtained was measured for different filtration times. Measured data were compared with the results calculated by the simulator, as depicted in Figure 8. The values obtained by the numerical model are in very good agreement with the experimental measured values. For example, after 24 h of shaping under a constant pressure of 3 × 10^6^ Pa, the pieces are approximately 110 mm thick, which was predicted by the simulator in the same conditions. One limitation appears in the results exposed in Figure 8. The casting kinetics are strongly dependent on the composition of suspensions. In fact, the suspension formulation controls their rheological behavior and cake porosity characteristics. As a consequence, the cake permeability can slightly vary, which strongly affects the casting kinetics. As an example illustrated in Figure 8 and Table 1 with 1 at.% Nd-doped suspensions, the cake permeability is increased by a factor of around 1.5 when the suspensions’ viscosity is increased by a factor of about 3 (i.e., when the PAA content is decreased from 1.0 to 0.7 wt.%). Consequently, the cake length is increased by a factor of about 2.4 for the same shaping time. This work shows that the model can predict casting kinetics with only the knowledge of cake microstructural characteristics, producing very accurate and reproducible tests.

### 3.2. Sintering Behavior of Green Compacts

This part aims to highlight the limits of the shaping process by pressure casting for the development of large transparent ceramics (i.e., with thickness > 10 mm). Indeed, the previous section showed that it was possible to manufacture large parts using this process. However, no published study in this field has focused on the influence of the thickness of the parts on their microstructural and optical homogeneity after sintering.

Ceramics of 1 at.% Nd:YAG were shaped by pressure slip-casting at a pressure of 30.10^5^ Pa in the form of pellets with a 50 mm diameter and 10 mm thickness and then sintered by natural sintering under vacuum at 1740 °C for 20 h at a heating rate of 5 °C·min^−1^. Suspensions used in this part were elaborated with optimized parameters of 30 vol.%, pH = 9, and 1.3 wt.% of PAA. The sintered samples obtained were characterized by SEM, optical microscopy, optical transmission, and laser efficiency.

**Figure 9:**
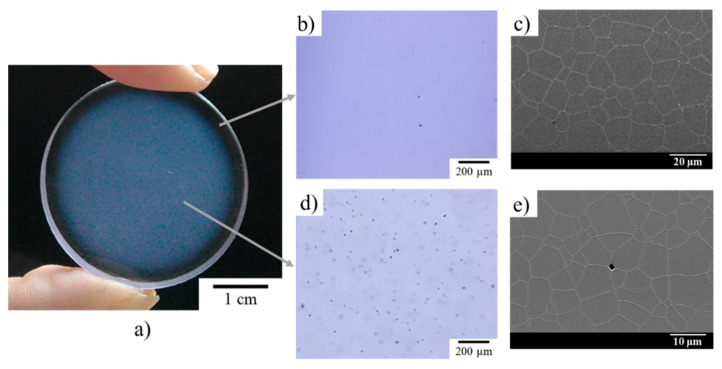
View of 1 at.% Nd:YAG ceramic with a 10 mm thickness obtained by pressure slip-casting and natural vacuum sintering at 1740 °C for 20 h. (**a**) Sample picture; Optical micrographs of (**b**) the shell and (**d**) the core; SEM micrographs of (**c**) the shell and (**e**) the core.

As illustrated in Figure 9a, the transparency of the ceramic is heterogeneous in the sample. A whitish haze is observed in the center of the sample, surrounded by a perfectly transparent ring. This difference is linked to the presence of light scattering defects located in the center of the samples. These defects are pores, as illustrated by the SEM micrographs in Figure 9c,e. These observations highlight an almost zero defect density on the edge of the sample (Figure 9b,c), while the hazy area in the center of the sample contains a much higher defect density (Figure 9d,e). From the analysis of SEM micrographs, the volume fraction of porosity present at the core of the material was estimated to be 0.019% for an average pore diameter in the order of 1.9 μm.

The mean grain diameter is 18.5 ± 0.5 µm and 6.6 ± 0.2 µm at the edge and in the core of the sample, respectively. Despite these differences, the grain size distribution remains homogeneous, regardless of the area observed, which indicates that the microstructures observed are the result of normal grain growth. Therefore, the differences in microstructure observed seem to indicate that the edge and core of the sample did not reach the same stage of densification. This observation highlights differential sintering between the edge and core of the sample. This phenomenon could be explained by the appearance of a thermal gradient within the material during the sintering cycle [42]. Indeed, during the rise in temperature, and due to the low apparent thermal conductivity of green ceramics, especially when they are porous and heated under vacuum [43], a decreasing thermal gradient between the surface and the core of the sample is imposed. The outside of the sample will therefore tend to densify before the core of the sample. This can generate a “skin” phenomenon, potentially slowing down or even stopping sintering in the core of the sample [44]. The higher the heating rate and the sample thickness are, the stronger the thermal gradient will be. The thickness of a perfectly dense sample that can be produced by natural sintering is therefore limited by this phenomenon. This may be avoided or at least mitigated with a slower temperature ramp (generally lower than 1 °C·min^−1^), but the obtained microstructure is generally coarser. A coarse microstructure of sintering ceramics generally leads to reduced mechanical properties. Therefore, here, the study on the sintering of Nd:YAG ceramics was focused on the application of pre-sintering, followed by densification post-treatment with HIP. In fact, applying external pressure at a high temperature can help remove porosity during the ultimate stage of densification.

**Figure 10:**
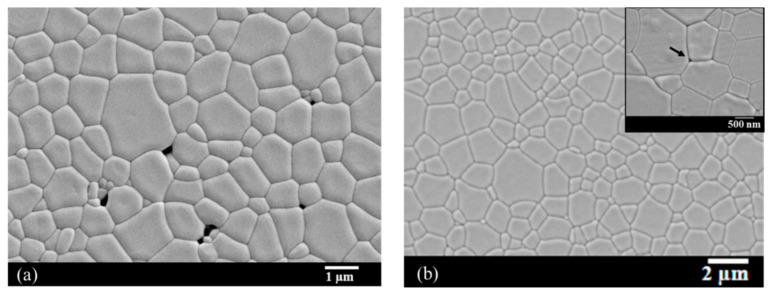
SEM micrographs of 1% Nd:YAG ceramics (**a**) pre-sintered under vacuum at 1625 °C for 5 h; and (**a**) after post-Hot Isostatic Pressing (HIP) at 1625 °C for 30 min under 190 MPa of argon gas. Insert at higher magnification in (**b**) shows a residual pore with about a 50 nm diameter (arrow) after post-HIP.

The samples were pre-sintered under vacuum at 1625 °C for 5 h. The relative density of the samples obtained is 99.1 ± 0.3% for an average grain diameter of 1.3 ± 0.1 µm and an average pore diameter of 0.37 ± 0.13 µm. The HIP post-treatment was carried out at 1620 °C for 30 min and under an argon pressure of 190 MPa. The corresponding microstructures before and after post-HIP are reported in Figure 10. These results show that the pressure applied during the HIP treatment is very effective in achieving total densification of the material at a limited temperature and in a limited time. Only a few rare intergranular pores of a very small size (i.e., 50 nm in diameter) are detected (see insert in Figure 10b).

**Figure 11:**
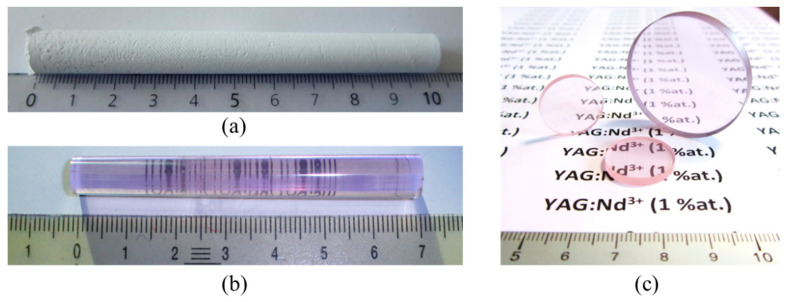
View of 1% Nd:YAG ceramics in the form of a barrel with a 100 mm length after shaping by pressure slip-casting (**a**), after presintering under vacuum + post-HIP at 1625 °C for 30 min under 190 MPa of argon (**b**), and in the form of discs of various sizes (**c**).

After post-HIP sintering treatment, the transparency of large samples (i.e., thickness > 10 mm) with a bar or pellet shape is homogeneous and of a good quality (Figure 11). These results show that pressure casting coupled with a post-HIP sintering technique seems to be a well-suited route for the manufacturing of large, homogeneous, and transparent parts.

**Figure 12:**
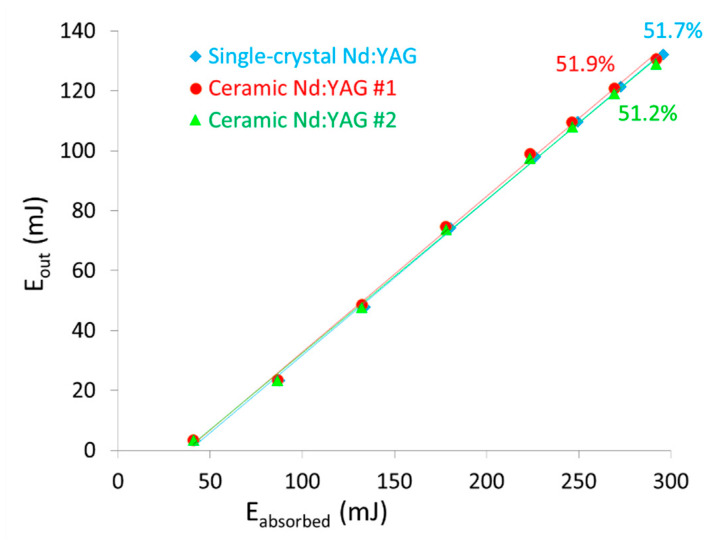
Laser efficiency at a 1064 nm output wavelength and under 808 nm diode pumping of 1 at.% Nd:YAG ceramics obtained by pressure slip-casting + post-HIP sintering treatment and an equivalent commercial standard single crystal.

Finally, after optical finishing (i.e., flatness < λ/10 and parallelism < 10 arc.sec), laser performance tests were carried out on the 2.5 and 15 mm diameter pellets in a cavity made up of two mirrors and diode pumping at 808 nm. The laser yield obtained is presented in Figure 12 and shows that the laser yield slope is equivalent to or even greater than that obtained for a single crystal of the same composition.

## 4. Conclusions

In this study, Nd:YAG transparent ceramics with a large size were elaborated by pressure slip-casting forming and reactive-sintering under vacuum, followed by HIP post-sintering techniques. Stable colloidal suspensions of primary oxides were obtained thanks to PAA addition. Cakes with a good homogeneity and fine microstructure were obtained. Simulation of the pressure slip-casting process with COMSOL^®^ software has shown that the cake permeability is a critical parameter that meanly controls the casting kinetics. Permeability can vary as a function of various process parameters, such as the slurry composition or viscosity. Finally, transparent Nd:YAG ceramics with a large size and a laser efficiency equivalent to that of a commercial single crystal were obtained.

## Figures and Tables

**Table 1:** Pressure slip-casting parameters and comparison of the calculated permeability with experimental values obtained for suspensions with 30 vol.% and pH = 9.

Composition (Primary Oxides Mixing)	PAA (wt.%)	η at 100 s^−1^ (mPa·s)	P (Pa)	ε (%)	d_p_ (10^−9^m)	k_Ergun_ (m^2^) *	k_Simul_ (m^2^) **
1 at.% Nd:YAG	1.3	18	1 × 10^6^	52	95	3.7 × 10^−17^	3.9 × 10^−17^
1 at.% Nd:YAG	1.3	18	3 × 10^6^	50	90	2.7 × 10^−17^	2.9 × 10^−17^
1 at.% Nd:YAG	1.0	35	3 × 10^6^	54	94	4.4 × 10^−17^	4.6 × 10^−17^
1 at.% Nd:YAG	0.7	100	3 × 10^6^	59	100	8.1 × 10^−17^	7.2 × 10^−17^
3.5 at.% Nd:YAG	1.6	19	3 × 10^6^	51	94	3.3 × 10^−17^	3.2 × 10^−17^
Undoped YAG	1.0	33	3 × 10^6^	55	94	4.8 × 10^−17^	4.6 × 10^−17^

* Permeability calculated from Equation (3) and experimental measurement of porosity characteristics (ε and *d_p_*) of the cake (see Figure 4 for *d_p_*). ** Permeability as the fitting parameter of the model implemented in COMSOL^®^ software to describe experimental casting kinetics.
